# Reversible Femtosecond Laser-Assisted Myopia Correction: A Non-Human Primate Study of Lenticule Re-Implantation after Refractive Lenticule Extraction

**DOI:** 10.1371/journal.pone.0067058

**Published:** 2013-06-24

**Authors:** Andri K. Riau, Romesh I. Angunawela, Shyam S. Chaurasia, Wing S. Lee, Donald T. Tan, Jodhbir S. Mehta

**Affiliations:** 1 Tissue Engineering and Stem Cell Group, Singapore Eye Research Institute, Singapore, Singapore; 2 Singapore National Eye Centre, Singapore, Singapore; 3 SRP Neuroscience and Behavioral Disorder Program, Duke-NUS Graduate Medical School, Singapore, Singapore; 4 Department of Ophthalmology, Yong Loo Lin School of Medicine, National University of Singapore, Singapore, Singapore; 5 Department of Clinical Sciences, Duke-NUS Graduate Medical School, Singapore, Singapore; 6 Ophthalmology Academic Clinical Program, Duke-NUS Graduate Medical School, Singapore, Singapore; University of Missouri-Columbia, United States of America

## Abstract

LASIK (laser-assisted in situ keratomileusis) is a common laser refractive procedure for myopia and astigmatism, involving permanent removal of anterior corneal stromal tissue by excimer ablation beneath a hinged flap. Correction of refractive error is achieved by the resulting change in the curvature of the cornea and is limited by central corneal thickness, as a thin residual stromal bed may result in biomechanical instability of the cornea. A recently developed alternative to LASIK called Refractive Lenticule Extraction (ReLEx) utilizes solely a femtosecond laser (FSL) to incise an intrastromal refractive lenticule (RL), which results in reshaping the corneal curvature and correcting the myopia and/or astigmatism. As the RL is extracted intact in the ReLEx, we hypothesized that it could be cryopreserved and re-implanted at a later date to restore corneal stromal volume, in the event of keratectasia, making ReLEx a potentially reversible procedure, unlike LASIK. In this study, we re-implanted cryopreserved RLs in a non-human primate model of ReLEx. Mild intrastromal haze, noted during the first 2 weeks after re-implantation, subsided after 8 weeks. Refractive parameters including corneal thickness, anterior curvature and refractive error indices were restored to near pre-operative values after the re-implantation. Immunohistochemistry revealed no myofibroblast formation or abnormal collagen type I expression after 8 weeks, and a significant attenuation of fibronectin and tenascin expression from week 8 to 16 after re-implantation. In addition, keratocyte re-population could be found along the implanted RL interfaces. Our findings suggest that RL cryopreservation and re-implantation after ReLEx appears feasible, suggesting the possibility of potential reversibility of the procedure, and possible future uses of RLs in treating other corneal disorders and refractive errors.

## Introduction

Myopia remains a significant ocular disability and economic burden by virtue of its high prevalence in most populations [1]. The prevalence of myopia has increased substantially in the past 50 years, possibly relating to environmental risk factors including increasing near work demands and reduced outdoor activities in children globally [2]–[4]. In combination with more sensitive pre-operative screening and wavefront-driven treatment profiles, the current generation of excimer laser platforms are safer, more precise, and more predictable than ever before, and the number of patients undergoing laser-assisted myopia treatment, in the form of laser in-situ keratomileusis (LASIK), has also increased rapidly over the last decade [5]. It has been estimated that more than 700,000 patients undergo LASIK annually in the United States alone and approximately 4 million LASIK surgeries are performed each year in China [6]–[8].

LASIK involves a 2-stage procedure, flap creation using either a microkeratome or increasingly more common, a femtosecond laser (FSL), followed by refractive removal of the anterior stromal by excimer stromal ablation, which is irreversible, and results in thinning of the central cornea [9], [10]. While LASIK remains a highly successful procedure, side effects such as post-operative glare and haloes, and dry eye symptoms have been documented [11]–[14]. The latter probably relating to a neurotrophic state due to transection of afferent sensory nerves in the anterior layers of the cornea stroma [14]. Biomechanical instability resulting from excessive stromal bed thinning can result in visual loss and significant ocular morbidity in the form of post-LASIK keratectasia in patients with undetected form fruste keratoconus or excessively thin residual stromal beds [15]. The incidence of corneal ectasia after LASIK is estimated at 0.2% to 0.6% [15], [16].

Refractive Lenticule Extraction (ReLEx; Carl Zeiss Meditec, Jena, Germany) is a recent alternative to LASIK surgery to correct myopia and astigmatism, which utilizes a single FSL platform without the need for an excimer laser [17]–[19]. ReLEx surgery involves the use of FSL to incise, and remove an aspheric, refractive lenticule (RL) of pre-determined power within the anterior corneal stromal layers, which results in corneal flattening, which in a similar manner to LASIK, corrects the eye’s refractive error.

In the original ReLEx procedure, Femtosecond Lenticule Extraction (FLEx) which essentially mimics a LASIK type procedure with the formation of an anterior hinged flap. The posterior surface of the lenticule is incised first (Figure S1A), followed by the anterior surface (Figure S1B), that extends beyond the limit of the posterior cut, which also forms the anterior corneal flap. The vertical edge of the hinged corneal flap is then cut, usually with a superior hinge, and the resultant anterior flap is then lifted aside, similar to a LASIK flap (Figure S1C). The RL, which conforms to an aspheric convex lens powered to the intended myopic correction, or is spherocylindrical in myopic astigmatic treatments, is then peeled away (Figure S1D), and after which the flap is repositioned (Figure S1E).

The more recent form of ReLEx however involves RL removal through a small pocket incision, without the formation of a LASIK-type flap, which provides greater tectonic and biomechanical stability as most of Bowman’s layer is no longer incised. SMILE or Small Incision Lenticule Extraction involves the creation of a sub-3 mm-incision to the surface, through which the RL is extracted. SMILE may also minimize damage to the sub-basal epithelial nerve plexus, thus possibly reducing post-operative dry eye symptoms, and also eliminating the risk of flap-related complications such as striae, and trauma-related flap dislocation.

A fully intact RL is the immediate by-product of ReLEx in all cases, and the concept of preserving this lenticule for either subsequent re-implantation into the same patient, or as allograft donor tissue in other patients, forms the basis of this research. In a previous study, we examined the viability of intrastromal keratocytes within the extracted RL after cryo-storage (28 days), and were able to show that the cells remained viable, undifferentiated, and expressed markers typical of keratocytes from fresh tissue [20]. In a separate proof of concept in vivo study using a rabbit model, we examined the feasibility of cryopreserving these lenticules and re-implanting them into the operated eye, to establish whether it was possible to restore stromal volume following previous myopic correction, and to evaluate the early tissue intergration and response to RL re-implantation [21]. We demonstrated that RL re-implantation restored pre-operative corneal thickness and caused minimal corneal haze and wound healing responses in the short term [21]. Implanted corneas were indistinguishable from un-operated control eye at 28 days post-re-implantation.

With the ever-increasing popularity of FSL-assisted myopia treatment today, the reversible technique that we describe in this study can have significant appeal to patients by offering them the reassurance of being able to restore their corneas to the pre-operative state and also allowing other future treatments. This study evaluated the long term tissue response associated with ReLEx surgery, and the safety, efficacy and long-term outcome of autologous, cryopreserved RL re-implantation following a myopic correction in a non-human primate model of ReLEx surgery, with an emphasis on determining the potential for reversibility with regards to restoration of corneal thickness, curvature and refractive status.

## Materials and Methods

### Animals

Twelve adult monkeys (*Macaca fascicularis*) underwent surgical procedures as depicted in Figure S2. Animals were housed in adjoining individual stainless steel monkey cages allowing social interactions. The cages were equipped with automatic watering systems. The room environment was continuously controlled for temperature (24±2°C), humidity (50±20%), light cycle (12 hours light:12 hours dark), and air change (10 to 15 air changes/hour). Food was withdrawn overnight prior to any anesthesia. Continuous clinical care (24 hours/7 days) was provided throughout the study to ensure prompt intervention when needed. Animals were anesthetized with 0.5 ml of ketamine hydrochloride (100 mg/ml intramuscularly; Parnell Laboratories, Alexandria, Australia) and 0.1 ml of medetomidine hydrochloride (1 mg/ml subcutaneously; Pfizer Animal Health, New York, NY) during ReLEx and lenticule re-implantation procedure, as well as during pre- and post-operative eye examinations. The monkeys were sacrificed under sedation at different time points (Figure S2) by overdose intravenous injection of sodium pentobarbitone (Jurox, Rutherford, Australia). At the conclusion of the study, a total of 8 eyes were used to study long-term effect of ReLEx (4 eyes for 8 weeks post-surgical study and the other 4 eyes for 16 weeks post-surgical study) and a total of 14 eyes were collected to study long-term effect of RL re-implantation (7 eyes for 8 weeks post-re-implantation study and the other 7 eyes for 16 weeks post-re-implantation study). The remaining unoperated eyes (n = 2) were used as controls for immunohistochemical analysis. All animals were treated according to the guidelines of the Association for Research in Vision and Ophthalmology’s Statement for the Use of Animals in Ophthalmic and Vision Research. The study protocol was approved by the Institutional Animal Care and Use Committee of SingHealth, Singapore.

### Refractive Lenticule Extraction (ReLEx) Procedure

ReLEx was performed using a VisuMax femtosecond laser system (Carl Zeiss Meditec). All experimental eyes underwent a spherical −6.00D myopia correction. The femtosecond laser parameters used in this experiment were as described previously [22], [23]: 120 µm flap thickness, 7.5 mm flap diameter, 175 nJ power, and side cut angles at 90 degrees. The spot distance and tracking spacing were set at 3 µm/3 µm for lenticule, 2 µm/2 µm for lenticule border, 3 µm/3 µm for flap, and 2 µm/2 µm for flap side cut. The diameter of the lenticule (equating to the optical zone) was 6.5 mm. Following the completion of the laser sequence, a Seibel spatula (Rhein Medical, Inc. Petersburg, FL) was inserted under the flap near the hinge and the corneal flap was lifted. The RL was gently undermined with the spatula and was then grasped with a forceps and extracted. The flap was finally repositioned and resulting flap striae was then smoothed out. A bandage contact lens (Bausch & Lomb, Rochester, NY) was placed over the flap and the eyelid was closed with a temporary tarsorraphy for 3 days using 6/0 silk suture.

### Storage and Re-implantation of Intrastromal Refractive Lenticule

The storage and cryopreservation of the extracted RL was conducted as described earlier [20], [21]. A marking indicating the 12 o’clock position of the cornea (hinge position) was first made on the RGP lens to indicate the corresponding anatomical position of the lenticule on the cornea before extraction. The lenticules were carefully transferred on to rigid gas permeable (RGP) contact lenses (Bausch and Lomb) with careful attention to maintaining anatomical lenticular orientation. The contact lens was placed in a lens case and the well was filled with a stock freezing solution containing 10% FBS (fetal bovine serum; Sigma, St. Louis, MO) and 20% dimethyl sulfoxide (DMSO; Sigma). Freezing of the RGP and contact lens case containing the lenticule was carried out at a controlled cooling rate within a cryo-container (“Mr. Frosty”, Thermo Fisher Scientific, Roskilde, Denmark) in a −80°C freezer overnight, and transferred into liquid nitrogen the following day for long-term storage.

**Figure 1:**
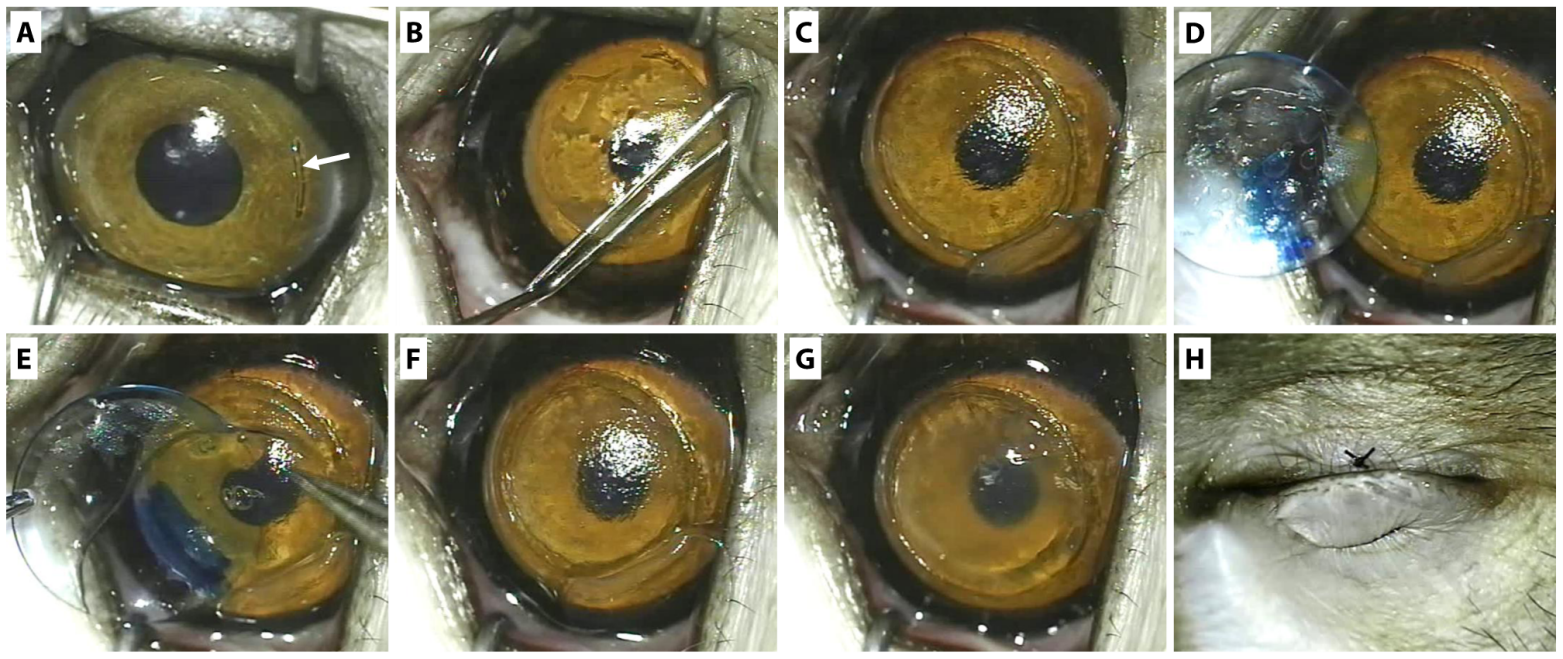
Photomontage of the refractive lenticule re-implantation procedure. (A) A small incision was first created near the hinge of the ReLEx flap (arrow). (B) This was followed by insertion of a Seibel spatula and flap flipper under the flap and by sweeping of the spatula under the flap to release the adhesion. (C) The stromal bed was exposed after lifting of the flap. (D) A blue marking at the 12 o’clock position on the rigid gas permeable (RGP) lens indicating the corresponding anatomical position of the lenticule on the cornea before extraction. (E) The lenticule was transferred onto the exposed stromal bed by sliding it from the RGP contact lens. (F) The lenticule on the stromal bed after a careful orientation and alignment. (G) The flap was eventually repositioned. (H) A bandage contact lens was then placed over cornea and the eyelid was closed with a temporary tarsorraphy for 3 days.

The re-implantation of the RL was performed on week 16 after ReLEx procedure. The RL and RGP lens were first allowed to warm to room temperature. After the monkeys were anesthetized, a Seibel spatula (Rhein Medical, Inc.) and flap flipper (Asico, Westmont, IL) was inserted through a small incision created near the hinge of the corneal flap (Figure 1A), and flap adhesions were slowly released by sweeping the spatula under the flap (Figure 1B). Upon the lifting of the flap (Figure 1C), the 12 o’clock orientation of the lenticule on the stromal bed was carefully observed (Figure 1D) before the lenticule was transferred directly onto the exposed stromal bed by sliding it from the RGP contact lens (Figure 1E and 1F). The flap was then repositioned centrally (Figure 1G) and a bandage contact lens (Bausch & Lomb) was placed over cornea and the eyelid was closed with a temporary tarsorraphy for 3 days (Figure 1H). Gentamicin sulphate (40 mg/ml; Shin Poong Pharmaceutical, Seoul, South Korea) and Dexamethasone sodium phosphate (4 mg/ml; Hospira, Lake Forest, IL) of 1 ml each were injected subconjunctivally following the re-implantation procedure. Prednisolone acetate (1%; Allergan, Irvine, CA) and Tobramycin (0.3%; Alcon, Fort Worth, TX) drops were administered 4 times a day for 1 week.

### Corneal Imaging: Slit Lamp Photography and Anterior Segment-Optical Coherence Tomography (AS-OCT)

Slit lamp photographs and AS-OCT scans were obtained pre-ReLEx and 3 days, and 2, 4, 8 and 16 weeks after ReLEx surgery. Similar time points were followed for RL re-implantation procedure. Slit lamp photographs were taken with a Zoom Slit Lamp NS-2D (Righton, Tokyo, Japan). Post-reimplantation corneal clarity was graded by using a method previously reported by Fantes et al. [24] Corneal cross-sectional visualization and measurement of corneal thickness were performed by using a Visante AS-OCT (Carl Zeiss Meditec). The examiner adjusted the system to position the vertex at the center of the AS-OCT image and then slowly moved the system away from the cornea until the vertical white beam was barely seen. Measurements of CCT were taken at the center (0.0 mm) and at 1 mm either side of the centre (+1.0 mm, −1.0 mm). The mean value of the three distances was then reported.

### Corneal Curvature and Spherical Error Measurement

Measurements of corneal curvature (keratometry) and spherical error (refractometry) were obtained pre-ReLEx, 8 and 16 weeks post-ReLEx and similarly followed for RL re-implantation. Keratometric values and spherical errors were measured using a Nidek ARK-30 Autorefractor/Keratometer (Hiroishi, Japan).

### *In vivo* Confocal Microscopy

In vivo confocal microscopy was performed pre-ReLEx, 3 days, and 2, 4, 8 and 16 weeks after ReLEx and lenticule re-implantation surgeries, using a Heidelberg retinal tomography HRT3 with Rostock corneal module (Heidelberg Engineering GmbH, Heidelberg, Germany). A carbomer gel (Vidisic; Mann Pharma, Berlin, Germany) was used as immersion fluid. All corneas were examined centrally with at least 3 z-axis scans epithelium to endothelium. In vivo confocal micrographs were then analyzed with the Heidelberg Eye Explorer version 1.5.1 software (Heidelberg Engineering GmbH). Semi-quantitative analysis of the reflectivity level of the lenticular anterior and posterior interface (in pixel) was performed using the Image J software as described previously [21], [22].

### Immunohistochemistry

After euthanization, the corneas were excised from the globe and embedded in Optimal Cutting Temperature (OCT) cryo-compound (Leica Microsystems, Nussloch, Germany). Frozen tissue blocks were stored at −80°C until sectioning. Serial sagittal corneal 10 µm sections were cut using a Microm HM550 cryostat (Microm, Walldorf, Germany). Sections were placed on polylysine-coated glass slides and air dried for 15 minutes.

Immunohistochemical staining was performed as described previously [21]–[23]. The following primary antibodies and the corresponding working dilution factor were used: mouse monoclonal antibody against cellular fibronectin (Millipore, Billerica, MA) diluted 1∶400; tenascin-C (Abcam, Cambridge, UK) diluted 1∶200; collagen type I (Sigma) diluted 1∶100; and CD18 (Novus Biologicals, Littleton, CO) diluted 1∶100 in the blocking solution. After washing with 1X PBS, the sections were incubated with goat anti-mouse Alexa Fluor 488-conjugated secondary antibody (Invitrogen) at room temperature for 1 hour. Slides were then mounted with UltraCruz Mounting Medium containing DAPI (Santa Cruz Biotechnology) and were observed and imaged with a Zeiss AxioImager Z1 fluorescence microscope (Carl Zeiss, Oberkochen, Germany).

Double immunohistochemical staining was performed as described previously [25], with minor modifications. Tissue sections were incubated with mouse monoclonal antibody against α-smooth muscle actin (α-SMA, Dako Cytomation, Glostrup, Denmark) diluted 1∶50 in the blocking solution, or with pre-diluted mouse monoclonal antibody against Ki-67 (Invitrogen, Carlsbad, CA) at 4°C overnight. On the following day, the sections were incubated with Alexa Fluor 488-conjugated secondary antibody (Invitrogen) at room temperature for 1 hour. After washing with 1X PBS, the sections were double stained with an Alexa Fluor 568-conjugated phalloidin probe (Invitrogen) at room temperature for 30 minutes. Slides were subsequently mounted with UltraCruz Mounting Medium containing DAPI (Santa Cruz Biotechnology) and observed with the Zeiss AxioImager Z1 microscope (Carl Zeiss).

### TUNEL Assay

To detect apoptotic cells, a fluorescence-based TUNEL assay (In Situ Cell Death Detection Kit, Roche Applied Science, Indianapolis, IN) was used according to the manufacturer’s instructions. Double staining with Alexa Fluor 568-conjugated phalloidin probe (Invitrogen) was then performed as described in the preceding sub-section.

### Statistical Analysis

Data were expressed as mean ± standard deviation. The p value was determined using the two-tailed Student's t-test with GraphPad Prism 5 (La Jolla, CA). Data were considered to be statistically significant when p<0.05.

## Results

### Slit Lamp Evaluation

**Figure 2:**
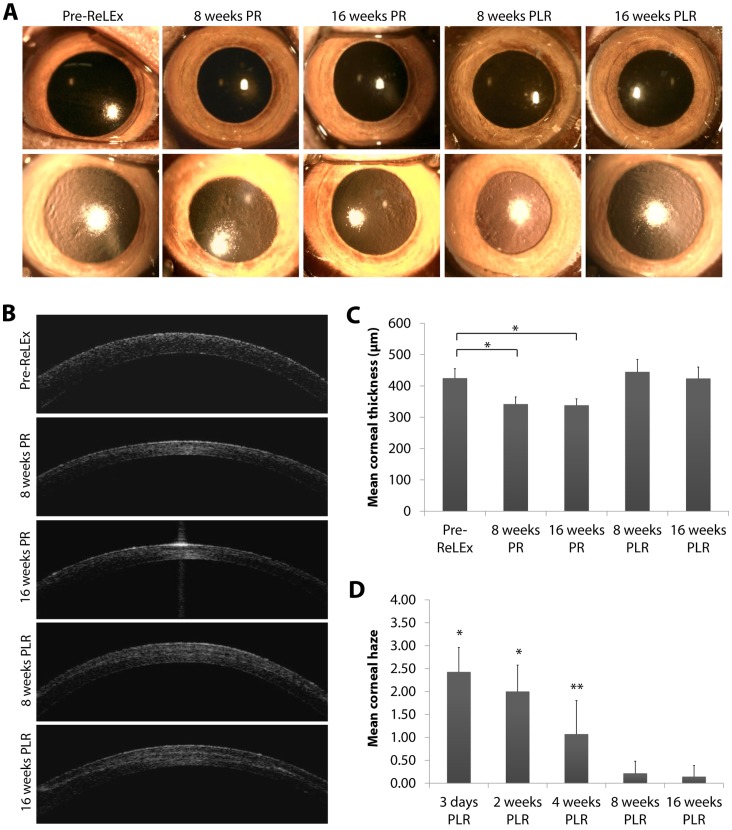
Slit lamp microscopy and anterior segment optical coherence tomography (AS-OCT) images of pre- and postoperative corneas. (A) Slit lamp (top panel) and retro illumination photographs (bottom panel) of the cornea before ReLEx and on week 8 and 16 after ReLEx and refractive lenticule re-implantation. (B) Temporal AS-OCT images of postoperative corneas shows thinning of the cornea after ReLEx and restoration of corneal thickness after lenticule re-implantation. (C) Mean corneal thickness before ReLEx and 8 and 16 weeks after ReLEx and lenticule re-implantation. (D) Post-reimplantation corneal haze graded based on observation of the slit lamp photographs found in Figure S3. Corneal clarity or haze is graded on a scale of 0–4 (from 0 being completely clear to 4 being completely obscured). Statistical significance was obtained by comparing post-operative to pre-operative corneal clarity. Error bars in the bar graphs represent standard deviation. Asterisk (*) and double asterisk (**) denote p<0.001 and p<0.05, respectively. PR: post-ReLEx, PLR: post-lenticule re-implantation.

Slit lamp examination with direct light and retro-illumination showed clear corneas at week 8 and 16 following ReLEx surgery with no stromal inflammation, epithelial ingrowth or diffuse lamellar keratitis (Figure 2A). Similar results were observed in the corneas at 8 and 16 weeks after RL re-implantation (Figure 2A). Post-re-implantation corneal clarity or haze was graded on a scale of 0 to 4 (from 0 being completely clear to 4 being completely obscured) as previously described [24], and was based on slit lamp photographs as shown in Figure S3. Corneal clarity was noted to progressively improve from 2.43±0.53 at day 3 after re-implantation, to 2.00±0.58 at week 2, to 1.07±0.73 at week 4, stabilizing to 0.21±0.27 and 0.14±0.24 at weeks 8 and 16, respectively (Figure 2D). There was no significant difference in the clarity of re-implanted corneas on week 8 and 16 compared to the pre-operated corneas.

### Restoration of Corneal Thickness

Anterior segment-optical coherence tomography (AS-OCT) scans showed thinner corneas after ReLEx surgery as expected, but the thickness was restored after RL re-implantation (Figure 2B). The anterior and posterior interface of the lenticule was visible on week 8 and 16 after re-implantation (Figure 2B). Central corneal thickness before surgery, 16 weeks post-ReLEx, and 16 weeks after RL re-implantation was measured at 425.05±30.25 µm, 338.33±20.41 µm, and 423.76±36.67 µm (n = 7), respectively (Figure 2C). There was a statistically significant difference in corneal thickness (p<0.001) between the corneas pre- and post-ReLEx surgery, but no significant difference between the corneas pre-ReLEx and post-lenticule reimplantation.

### Restoration of Corneal Curvature and Spherical Error

Corneal curvature (keratometry) measurements suggested an obvious flattening of the cornea consistent with the −6.00D myopia treatment on week 8 and 16 after ReLEx procedure (Table 1). On week 8 and 16 after re-implantation of the RL, corneas were steepened centrally and the keratometry values were similar to the pre-operative corneas. Similar observations were made in relation to changes in corneal spherical error (Table 1). There were no significant differences in the spherical error values in the pre-ReLEx and post-re-implantation corneas.

**Table 1:** Mean corneal curvature measured by keratometer and mean spherical error measured by refractometer (n = 7).

	Keratometry (D)^c^	*p* value^d^	Sphericalerror (D)^c^	*p* value^e^
Pre-ReLEx	58.6±2.1		−1.64±0.56	
8 weeks PR^a^	54.5±1.3	0.001	+4.43±0.87	<0.001
16 weeks PR^a^	54.1±2.4	0.003	+4.29±0.86	<0.001
8 weeks PLR^b^	57.9±0.8	0.438	−1.61±0.43	0.895
16 weeks PLR^b^	58.0±1.2	0.506	−1.64±0.35	0.891

a PR = post-ReLEx.

b PLR = post-lenticule re-implantation.

c D = diopter.

d *p* values relative to the keratometry before ReLEx.

e *p* values relative to the spherical error before ReLEx.

### In vivo Confocal Microscopy

**Figure 3:**
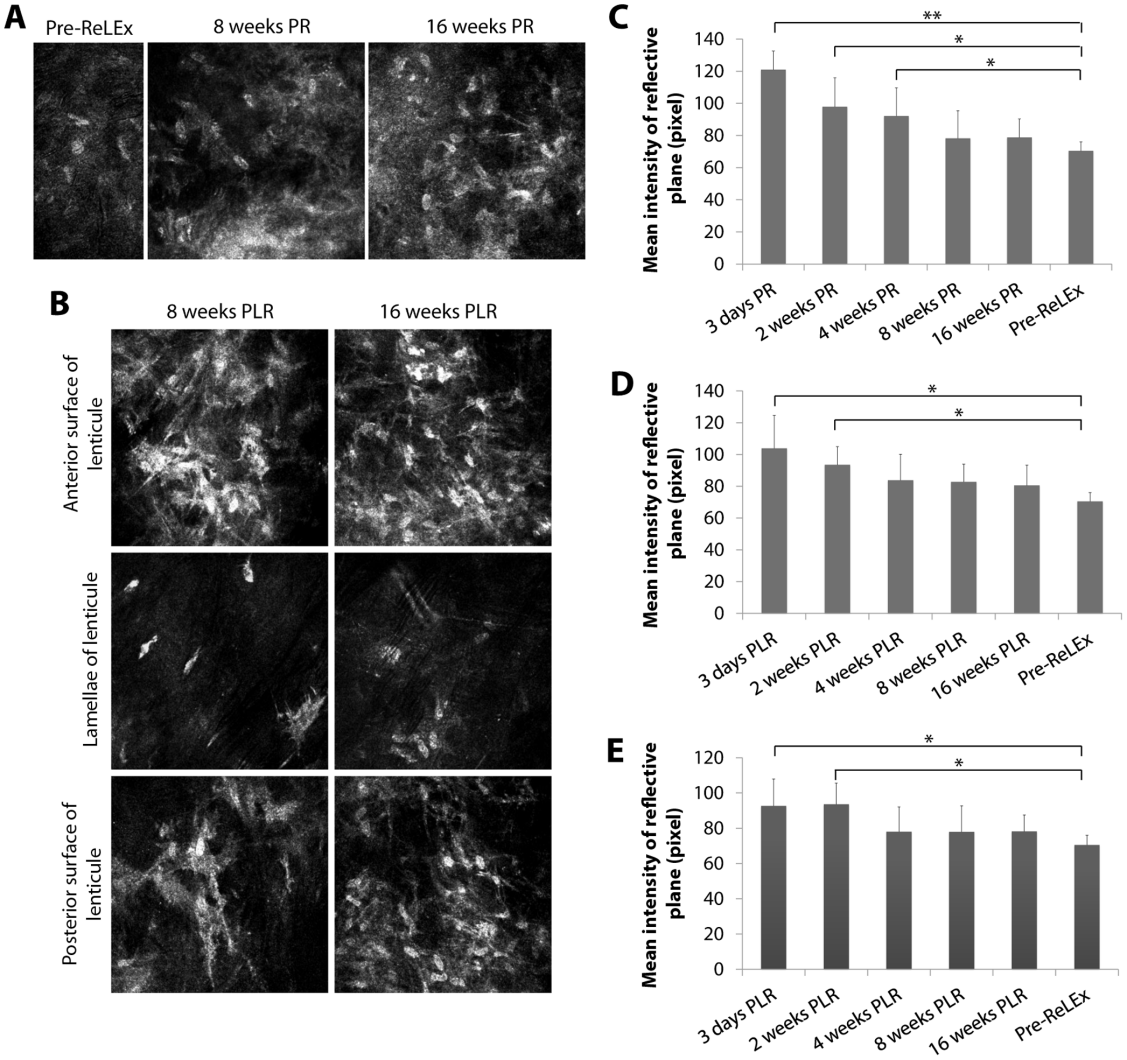
In vivo confocal micrographs of pre- and post-operative corneas. **(**A) Horizontal surgical plane between the flap and stromal bed on week 8 and 16 after ReLEx. This region is normally indicated by a relatively higher light reflective layer. Keratocyte repopulation could be observed on week 8 and 16. (B) The top panel shows the anterior interface of the re-implanted lenticule. The middle panel shows the presence of keratocytes within the lamellae of the lenticule and the bottom panel shows the posterior interface of the lenticule. Keratocyte repopulation of anterior and posterior borders of the lenticule occurred by week 8 after lenticule re-implantation. (C) Mean reflectivity level of the laser incision site on day 3, and 2, 4, 8 and 16 weeks after ReLEx. The representative in vivo confocal images of the cornea at the stated time point can be found in Figure S4A. (D) Mean reflectivity level of the re-implanted lenticule’s anterior interface on day 3, and 2, 4, 8 and 16 weeks. (E) Mean reflectivity level of the lenticule’s posterior interface post-reimplantation. The representative in vivo confocal images of the cornea at the stated time point in panes D and E can be found in Figure S4B. Error bars in the bar graphs represent standard deviation. Asterisk (*) and double asterisk (**) indicate p<0.05 and p<0.001, respectively. PR: post-ReLEx, PLR: post-lenticule re-implantation.

In vivo confocal photographs showed appearance of light reflective layer or haze in the femtosecond laser incision or stromal injury plane. Post-ReLEx surgical regions were marked by interspersed small particles with variable size and reflectivity, which were likely to be mixture of post-surgical debris, inflammatory cells and disrupted extracellular matrix (Figure S4A). The reflectivity level was gradually decreased in the subsequent follow-ups (Figure 3A and S4A) and quantified (Figure 3C). The intensity level of the reflective layer decreased from 120.99±11.57 pixels on post-operative day 3 to 92.11±17.52 pixels on week 4, and to 78.79±11.54 pixels on week 16. There were significant differences observed between post-operative day 3 and pre-operative corneas (p<0.001), as well as between week 2 and pre-operative corneas (p<0.001) and between post-operative week 4 and pre-operative corneas (p<0.05). In addition, keratocyte re-population was seen on week 8 and 16 at the flap interface of post-ReLEx corneas (Figure 3A).

Similar to post-ReLEx corneas, the anterior (top panel) and posterior border (bottom panel) of the RL showed a higher level of light reflectance and was acellular in the earlier time points after re-implantation (Figure S4B). The reflectivity level was progressively reduced in the subsequent follow-ups (Figure 3B and S4B) and is depicted in bar graphs (Figure 3D and 3E). The intensity levels of the reflective layer or haze at the lenticular anterior border decreased from 103.93±20.71 pixels on post-reimplantation day 3 to 83.84±16.27 pixels on week 4, and to 80.57±12.78 pixels on week 16. Similarly, the reflectance at the lenticular posterior interface reduced from 92.63±15.32 pixels on day 3 to 78.03±14.08 pixels on week 4, and to 78.20±9.31 pixels on week 16. There were statistically significant differences observed between day 3 and control corneas (p<0.05), and between week 2 and control corneas (p<0.05) at both interfaces. Keratocytes were visible at both interfaces of the re-implanted lenticule on week 8 and 16 (Figure 3B). Activated and elongated keratocytes within the center of the lenticule were observed on week 8 (Figure 3B, middle panel). At week 16, most of the keratocytes appeared normal and quiescent (Figure 3B, middle panel), and resembled those found in the pre-operative corneal stroma (Figure 3A, control).

### Immunohistochemical Analysis

**Figure 4:**
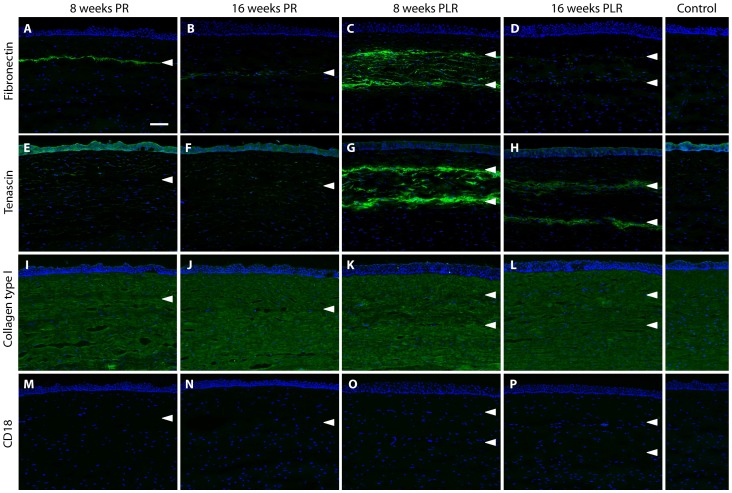
Expression of fibronectin, tenascin, collagen type I and CD18 in post-operative central corneas. (A–D) Fibronectin predominantly appeared along the laser incision site or lenticular interface. The expression was reduced over time after either ReLEx or refractive lenticule re-implantation. (E–H) Tenascin was absent along the flap interface on week 8 and 16 following ReLEx, but was present along the borders of the stromal lenticule after re-implantation. The intensity of the staining was attenuated over time. (I–L) Collagen type I was expressed uniformly in the full thickness of corneal stroma. No significant anomaly in collagen arrangement was observed in the corneas post-ReLEx and post-reimplantation. (M–P) CD18-positive cells were not seen in all post-operative corneas. Unoperated corneas were used as control. Arrowheads indicate the location of the laser incision site or lenticular interface. PR: post-ReLEx, PLR: post-lenticule re-implantation. Scale bar: 50 µm.

On week 8 post-ReLEx surgery, expression of fibronectin was observed along the laser incision site (Figure 4A) and which was weaker on week 16 (Figure 4B). Similar phenomenon could be seen in the corneas after refractive lenticule re-implantation. Fibronectin was predominantly present along the anterior and posterior interface of the re-implanted lenticule on week 8 (Figure 4C). Its expression was reduced over time and weaker staining was detected on week 16 (Figure 4D). Tenascin is normally found in the corneal epithelial cells and only found in the corneal stroma after an injury [26]. On week 8 and 16 after ReLEx, tenascin was no longer expressed along the laser injury plane (Figure 4E and 4F). Similar to fibronectin, tenascin was predominantly present along the anterior and posterior interface of the re-implanted lenticule on week 8 (Figure 4G) and its expression was diminished by week 16 (Figure 4H).

Collagen type I, the predominant collagen type presents in the cornea stroma [27], [28], was uniformly expressed in full thickness of the stroma of control corneas and all the samples in the post-operative corneas. No significant alteration to the collagen expression was observed after either ReLEx procedure (Figure 4I and 4J) or RL re-implantation (Figure 4K and 4L). Leukocyte integrin β2 (CD18), an inflammatory marker and mediator of polymorphonuclear leukocyte (PMN) migration within the corneal stroma [29], was not expressed in post-ReLEx (Figure 4M and 4N) and post-re-implantation corneas (Figure 4O and 4P).

**Figure 5:**
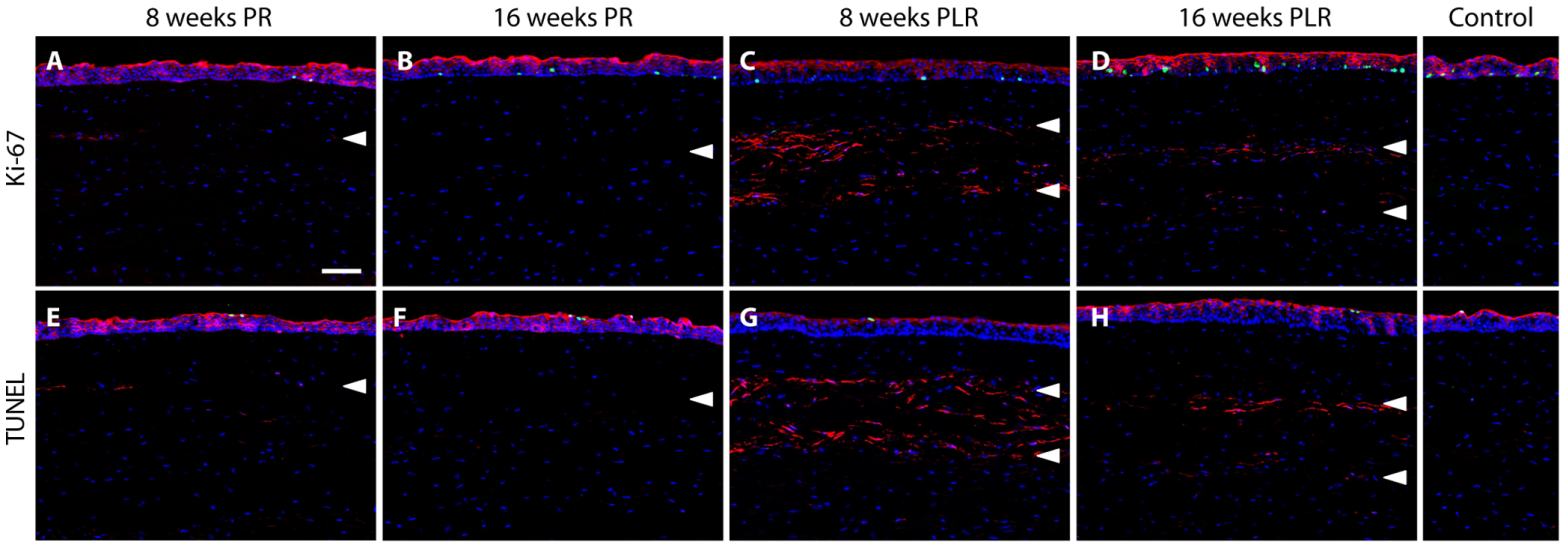
Immunofluorescent staining of Ki-67, TUNEL and phalloidin in post-operative central corneas. (A–D) Ki-67-positive cells (green) were not found in the corneal stroma on week 8 and 16 after ReLEx and refractive lenticule re-implantation. (E–H) Similarly, presence of TUNEL-positive cells (green) was also not detected in the corneal stroma. In pane A–H, F-actin marker (red), phalloidin, was observed in the laser incision site or lenticular interface. Its presence was attenuated over time. Nuclei were counterstained using DAPI (blue). Unoperated corneas were used as control. Arrowheads indicate the location of the laser incision site or lenticular interface. PR: post-ReLEx, PLR: post-lenticule re-implantation. Scale bar: 50 µm.

In all post-ReLEx and RL re-implanted corneas, no proliferating Ki-67-positive cells were observed within the stroma (Figure 5A–5D) and no apoptotic TUNEL-positive cells were found within the lenticule or stroma (Figure 5E–5H). Cell migration, indicated by the relatively strong staining of phalloidin suggest the intracellular assembly of F-actin in the earlier time points, which later decreased after ReLEx surgery (Figure 5A,B and 5E,F). However, presence of phalloidin could be seen within the re-implanted lenticule and was abundant in the anterior and posterior portion of the lenticule on week 8 post-re-implantation (Figure 5C and 5G), although the presence of F-actin became less abundant on week 16 (Figure 5D and 5H).

**Figure 6:**
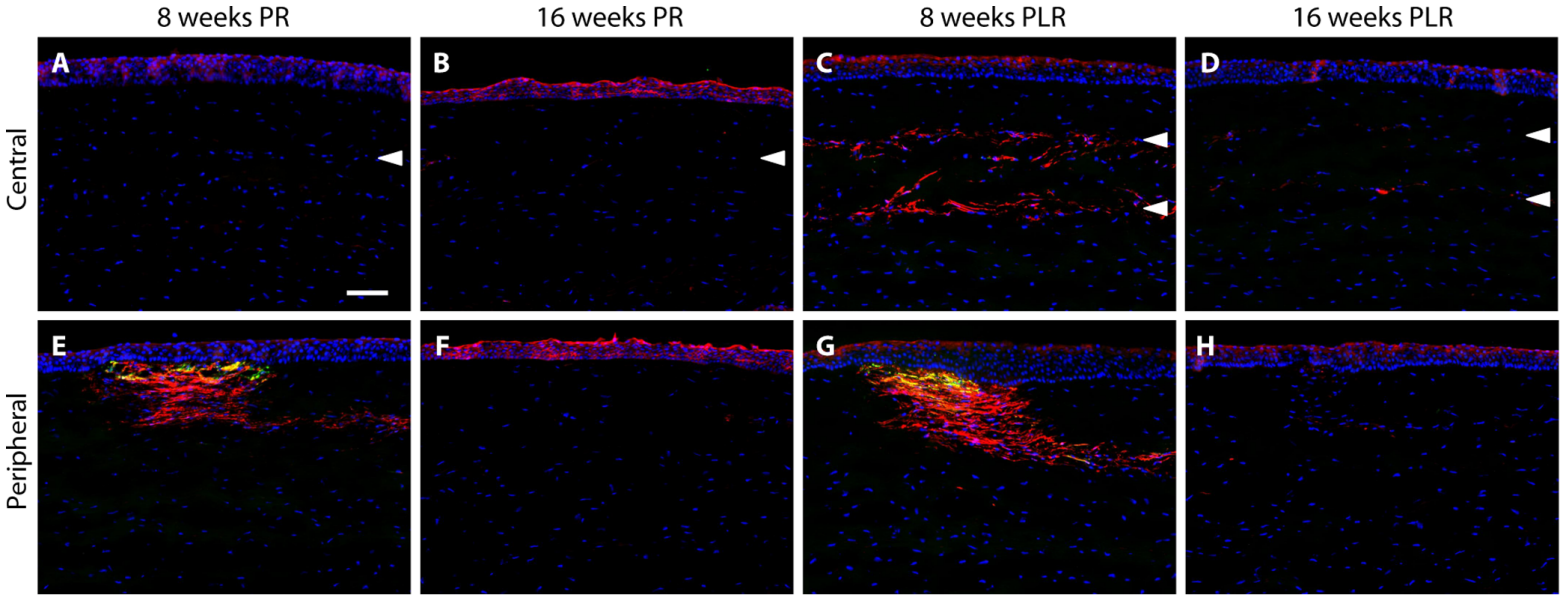
Expression ofα-smooth muscle actin (α-SMA) in the post-operative central corneas and peripheral flaps. (A–D) α-SMA (green), a marker of myofibroblasts, was not present in the central corneas on week 8 and 16 after both ReLEx and refractive lenticule re-implantation. (E–H) α-SMA (green) was expressed at the flap periphery and co-localized with F-actin (red) subepithelially on week 8 post-ReLEx and lenticule re-implantation, but was absent 16 weeks after both surgical procedures. In pane A–H, α-SMA (green) was double immunostained with F-actin marker (red), phalloidin. Nuclei were counterstained using DAPI (blue). Arrowheads indicate the location of the laser incision site or lenticular interface. PR: post-ReLEx, PLR: post-lenticule re-implantation. Scale bar: 50 µm.

There were no myofibroblasts detected at the central cornea on week 8 and 16 after ReLEx (Figure 6A and 6B) and RL re-implantation (Figure 6C and 6D), which was indicated by the absence of α-smooth muscle actin (α-SMA) expression. α-SMA, which has been reported to be present at LASIK flap margin due to the incision of the epithelial basement membrane [30], was detected only subepithelially and co-localized with the F-actin at the corneal flap edge on week 8 following ReLEx (Figure 6E) and RL re-implantation (Figure 6G). On week 16, myofibroblasts were absent in post-ReLEx (Figure 6F) and RL re-implantation groups (Figure 6H).

## Discussion

All forms of laser vision correction on the cornea currently involve removal of the anterior layers of the cornea, which may affect structural integrity and stability. The clinical implication of a reversible refractive procedure would be to restore corneal volume and possibly corneal integrity in patients, who may develop corneal ectasia following a refractive procedure such as LASIK, perhaps with the addition of collagen cross-linking to further strengthen the cornea [31]. Restoration of corneal stromal volume could also provide the opportunity for further refractive correction to improve vision [32], [33]. This study suggests that anatomical restoration of the cornea by lenticule re-implantation following ReLEx surgery is a viable option. Another application of the findings from this study could be the treatment of presbyopia, in emmetropic presbyopes or in post-refractive surgery emmetropes by the use of autologous or allograft corneal stromal lenticules acting as a stromal refractive inlay.

This study addresses two aspects of ReLEx surgery, namely, the intermediate-term corneal tissue response to the ReLEx procedure, and the intermediate-term feasibility of ReLEx lenticule re-implantation. With regards to the long-term evaluation of the tissue responses to ReLEx surgery, we observed only a mild corneal wound healing reaction, with minimal inflammatory cellular and myofibroblast responses, and normal collagen type I expression in the monkeys up to 16 weeks post-ReLEx. In addition, slit lamp examination and in vivo confocal microscopy revealed virtually no corneal haze formation. We have further demonstrated the medium-term feasibility of RL re-implantation post-ReLEx surgery in an in-vivo non-human primate model. The effectiveness of this technique in reversing the refractive procedure was demonstrated by the restoration of corneal thickness, curvature and refractive error indices to near pre-operative values following RL re-implantation. There was similarly minimal inflammatory response, myofibroblastic formation, and wound healing reaction, or no abnormal collagen type I expression after 8 weeks. In addition, we noted keratocyte re-population along the re-implanted RL interfaces.

With regards to corneal morphology, evaluation of the 3 ocular biometrics of corneal thickness (Figure 2C), corneal curvature (keratometry; Table 1) and refractive state (spherical error; Table 1) measured before and after ReLEx surgery, and after the RL re-implantation confirmed that restoration of all 3 parameters were achieved after RL re-implantation. Pre-operative corneal thickness was measured at 425.05±30.25 µm and was reduced 16 weeks post-ReLEx at 338.33±20.41 µm (p<0.001). Corneal thickness was restored to near pre-operative values after the RL re-implantation (423.76±36.67 µm on week 16 post re-implantation). Similarly, refraction and topography were restored after RL re-implantation. Corneal keratometry pre-ReLEx was 58.6±2.1D and became flatter 16 weeks after ReLEx (54.1±2.4D; p<0.05), but was restored to 58.0±1.2D at week 16 after RL re-implantation (p = 0.506). The spherical error before ReLEx was −1.64±0.56D, becoming +4.29±0.86D at week 16 after −6.00D myopic correction (p<0.001), which indicates that the eyes were −0.07±0.45D from the intended correction. This result was comparable to that reported in patients 3 months post-FLEx in our earlier study (0.33±0.50D) [19]. The refraction was restored to −1.64±0.35D at week 16 after RL re-implantation (p = 0.891). These data, which shows that LR re-implantation can indeed lead to full restoration of the anatomical changes which occurred after ReLEx surgery, confirms that clinical reversibility of the refractive procedure is possible. The keratometric values being 0.6D less than pre-operative values were possibly due to the formation of a corneal flap. This may be obviated when reversing a SMILE procedure.

Slit lamp examination in the re-implanted corneas showed mild haze in the first 2 weeks after surgery, but transparency progressively improved at subsequent follow-up time points. These observations were matched by a commensurate reduction in the levels of interface reflectivity seen during in vivo confocal microscopy. The absence of myofibroblasts (α-smooth muscle actin expressing cells) in the central cornea further confirms our results obtained with slit lamp and in vivo confocal microscopy in terms of haze, usually attributed to these cells. In vivo confocal analysis also showed quiescent keratocytes within the lenticular lamellae and later, re-population of the anterior and posterior lenticular interface by week 4 after re-implantation, suggesting the cornea’s attempt to return to a quiescent state. In the present study, the re-population appeared to occur through migration of adjacent keratocytes as indicated by F-actin staining, rather than by cell proliferation, as indicated by the absence of Ki-67-positive cells in the stroma. This is markedly different from pathological responses seen after excimer laser ablation in PRK and LASIK where the keratocytes are decellualrized [34]. Keratocytes, seen within the re-implanted lenticule, were probably cells that have survived the process of cryopreservation. As we have shown in our earlier study [20], these cells remain viable in cell culture after 1-month cryopreservation and also express keratocyte markers. The presence of these keratocytes along the lenticular borders, as well as within the lenticule is important in regulating the post-reimplantation wound healing [35]. In addition, keratocytes have been shown to be responsible in modulating extracellular matrix synthesis and cytoskeleton organization, which contribute to the mechanical strength of a collagen-based biomaterial [36]. This finding presents the fundamental implication of the ReLEx reversal technique, particularly in treating biomechanically compromised ectatic corneas.

The RL re-implantation didn’t appear to induce any significant alteration in wound healing reaction (low level of fibronectin) or incite an inflammatory (absence of CD18 expression) response. In addition, the uniform presence of collagen type I throughout the full-thickness of post-re-implanted corneal stroma indicates normal collagen expression, maintaining corneal transparency.

Parallels may be drawn between ReLEx lenticule re-implantation and epikeratophakia. Epikeratophakia was earlier described as a potential treatment for myopia and other refractive states such as aphakia [37], but was also used as a method for stromal volume restoration in the treatment of keratoconus [38]. Epikeratophakia involved the removal of host corneal epithelium and suturing a cryolathed donor corneal lenticule onto Bowman’s membrane, as an overlay allograft with tucked in edges at the trephination margins, over which host epithelium would heal, but this procedure was ultimately abandoned due to imprecise refractive outcomes arising from inadequate technological advances in lamellar donor preparation, and also onset of interface scarring, and poor visual outcomes partly related to the fact that this was essentially a surface-based procedure and subject to epithelial surface wound healing and epithelial-stromal inflammatory reactions, partly also exacerbated by the need to suture the overlaid lenticules [37]–[40]. In our technique of RL re-implantation, the absence of sutures and the deeper approach of lenticule placement beneath a corneal flap obviate much of the inflammatory and wound healing responses seen in epikeratophakia, which we have now shown to be minimal in this ReLEx reversal technique. The success of LASIK over the older excimer procedure of photorefractive keratectomy (PRK), was predicated on a much more predictable and minimal wound healing response when laser ablation was performed intrastromally beneath the LASIK flap, which largely obviated issues of epithelial healing and the potential for myopic regression which occurred with PRK. LASIK therefore became a much more predictable refractive procedure, which no longer requiring several weeks of topical steroids to prevent myopic regression, haze and scarring inherent in PRK. This is quite analogous to the comparison between epikeratophakia and RL re-implantation technique, the former being a surface-related wound healing procedure, and RL re-implantation being an intrastromal procedure with minimal wound healing responses.

Post-LASIK ectasia can be caused by the pre-existing corneal disease, for example undetected forme fruste keratoconus and/or biomechanical instability triggered by excessive stromal removal leading to weakening of the residual corneal stromal bed as a result of flap creation and excimer laser ablation. A common strategy to prevent post-LASIK ectasia is by empirically ensuring a residual stromal bed of 300 µm or greater, predicated on the perceived higher incidence of keratectasia which occurred in the earlier years of LASIK surgery, when 200 µm was suggested to be the minimal bed thickness. However, ensuring a minimal residual bed thickness of 300 µm does not totally prevent the occurrence of corneal ectasia [41]. Current clinical methods for stromal volume restoration for the treatment of corneal ectasia are limited to relatively invasive surgical approaches such as anterior lamellar keratoplasty (ALK), which involves surgical dissection and replacement of part of host corneal stroma with donor stromal tissue, or even full thickness penetrating keratoplasty (PK) [42]. Keratoplasties have significant complications which include the need for corneal sutures (with suture-related complications), and totally unpredictable induction of significant degrees of refractive errors, including high astigmatism, which would not be an acceptable outcome to refractive surgery patients who sought emmetropia in the first instance [43]. In contrast, a reversible refractive procedure described in the present study has significant advantages in terms of accurate FSL lamellar excision and precise refractive correction. The RL insertion technique is also relatively easier to perform surgically and could potentially be performed by refractive surgeons experienced in LASIK or ReLEx surgery. Moreover, the use of patient’s autologous stromal tissue avoids the risk of graft rejection, which can still occur in the form of epithelial or stromal rejection in ALK, and obviously can occur in the form of endothelial rejection with significant graft failure rates in PK surgery [44]. This technique may potentially be further improved by stabilizing the RL and/or the host cornea biomechanically by collagen cross-linking [31].

The onset of presbyopia remains a major visual disability for the refactive surgery patient, who previously enjoyed unaided distance vision prior to the onset of presbyopia. Current presbyopic treatment options for these patients include monocular implantation of a presbyopic intrastromal inlay, such as the Kamra (AcuFocus, Irvine, CA) or PresbyLens (ReVision Optics, Lake Forest, CA), both of which are currently undergoing U.S. Food and Drug Administration clinical trials; Laser Blended Vision (Carl Zeiss Meditec); and refractive lens exchange. However, biocompatibility related complications of non-biological implants have been reported in the literature, which included alterations in tear film thickness and corneal topography [45], corneal erosions [46], and peri-inlay deposits [47]. Corneal stromal restoration through RL re-implantation presents the opportunity for restoring the myopic status in the non-dominant eye to previous low myopia, thus resulting in monovision [48]. An alternative strategy is the possibility of re-implantation of a smaller autologous RL, reshaped to a positive refractive power (ie. +1.00 or +3.00D). In this scenario, the cryopreserved RL could act as an autologous, biological intrastromal inlay, similar to the polymeric corneal refractive inlays currently used commercially for presbyopic treatment, but with clear advantages in terms of biocompatibility and tissue integration. The relative absence of inflammation and wound healing processes which we have shown in this study would suggest that the various complications of scarring, haze and corneal melting seen in non-biological presbyopic implants would largely be obviated.

There are a few limitations in the RL collection and cryopreservation method that needs to be addressed before its application for clinical use. For storage of the RL, we utilized a simple rigid gas permeable (RGP) contact lens with an orientation mark indicating the 12 o’clock position of the lenticule relative to the eye before the ReLEx surgery. This approach sufficed for the present study where the attempted refractive correction was purely spherical and non-toric. In a clinical setting where patients are likely to have sphero-cylindrical toric corrections, orientation of the lenticule may be more critical, and a lenticular storage container would need to be designed to correctly indicate axial RL orientation. For the concept of reshaping RLs to a small, spherical, small optic presbyopic lenticule, obviously reshaping approaches using excimer or femtosecond laser technologies, or cryolathing, will need to be developed, and also take into account the refractive status of the original RL utilized. For allografts RLs, prior informed consent from patients who have donated their RL, and also a full serological evaluation of potential donors similar to that required in eye bank cornea tissue donors for corneal transplantation would be required.

For cryopreservation of the RL, we utilized dimethyl sulfoxide (DMSO), a commonly used non-toxic cryoprotectant, in order to prevent cellular damage during the freezing in liquid nitrogen. DMSO has previously been used in the storage of cord blood-derived stem cells [49]. Using the same cryo-storage method, we found tissue edema after 1-month cryopreservation, likely due to altered fluid balance within the lenticule [20]. The swelling of the RL reduced corneal clarity and caused corneal edema for a week after re-implantation in rabbits [21]. We observed similar findings in the primate model described in this study. A similar period of reduced corneal clarity may be expected in humans. Alternative techniques of cryopreservation, e.g. vitrification technique have been studied in the cornea previously [50], [51]. Vitrification was found to be effective in preventing ice crystal formation and disruption of collagen arrangement. Vitrification may be employed for the cryo-storage of patients RL, and further studies are required to optimize long term cryo-storage of RLs.

In conclusion, we have demonstrated the reversibility of a FSL-assisted myopia treatment (ReLEx) using a non-human primate model of refractive surgery. This study for the first time demonstrates that a laser refractive corneal procedure can be safely and effectively reversed. The potential option of RL cryo-storage after ReLEx allows refractive patients to preserve their tissue for subsequent re-implantation in the event of keratectasia, or a change in their refractive state, including presbyopia, or to donate their RLs for other patients to treat these same conditions and other forms of keratectasia, including keratoconus.

## Supporting Information

Figure S1 **Photomontage of refractive lenticule extraction (ReLEx) procedure.** After fixation of cornea under the suction cone, the femtosecond laser first creates the posterior surface of the lenticule centripetally (A), followed by the anterior surface of the lenticule centrifugally (B). The resultant anterior flap is then lifted (C), similar to a LASIK flap, and the refractive lenticule is manually removed (D). The flap is finally repositioned (E).
(TIF)

Figure S2 **Flowchart showing the time points at which the samples were collected: ReLEx or lenticule re-implantation was performed during the study.** RE and LE denote right eye and left eye, respectively. ReLEx is the abbreviation of Refractive Lenticule Extraction.
(TIF)

Figure S3 **Slit lamp and retro illumination photographs of the post-reimplantation corneas.** Slit lamp (top panel) and retro illumination photographs (bottom panel) of the cornea before ReLEx and on day 3, week 2, 4, 8 and 16 after refractive lenticule re-implantation. On day 3, the cornea appeared hazy with appearance of post-surgical debris. The appearance of haze was reduced in the subsequent follow-ups and was absent by week 8 and 16. PLR: post-lenticule re-implantation.
(TIF)

Figure S4 **In vivo confocal micrographs of the pre- and post-operative corneas.** (A) Horizontal surgical plane between the flap and stromal bed on day 3, weeks 2, 4, 8 and 16 after ReLEx. This region is normally marked by the presence of light reflective particles (haze), which gradually decreased in intensity over time. Keratocyte re-population could be observed on week 8 and 16. (B) The top panel shows the anterior interface of the re-implanted refractive lenticule. The middle panel shows the presence of keratocytes within the lamellae of the lenticule and the bottom panel shows the posterior interface of the lenticule. The intensity of the reflective layer observed in both interfaces was attenuated over time. Keratocyte re-population of anterior and posterior borders of the lenticule occurred by week 8 after lenticule re-implantation. PR: post-ReLEx, PLR: post-lenticule re-implantation.
(TIF)
