# Supplementary figures and images for: Reversible Femtosecond Laser-Assisted Myopia Correction: A Non-Human Primate Study of Lenticule Re-Implantation after Refractive Lenticule Extraction
Source: PLoS One. 2013 Jun 24;8(6):e67058. doi: 10.1371/journal.pone.0067058 (PMC3691223; doi:10.1371/journal.pone.0067058)

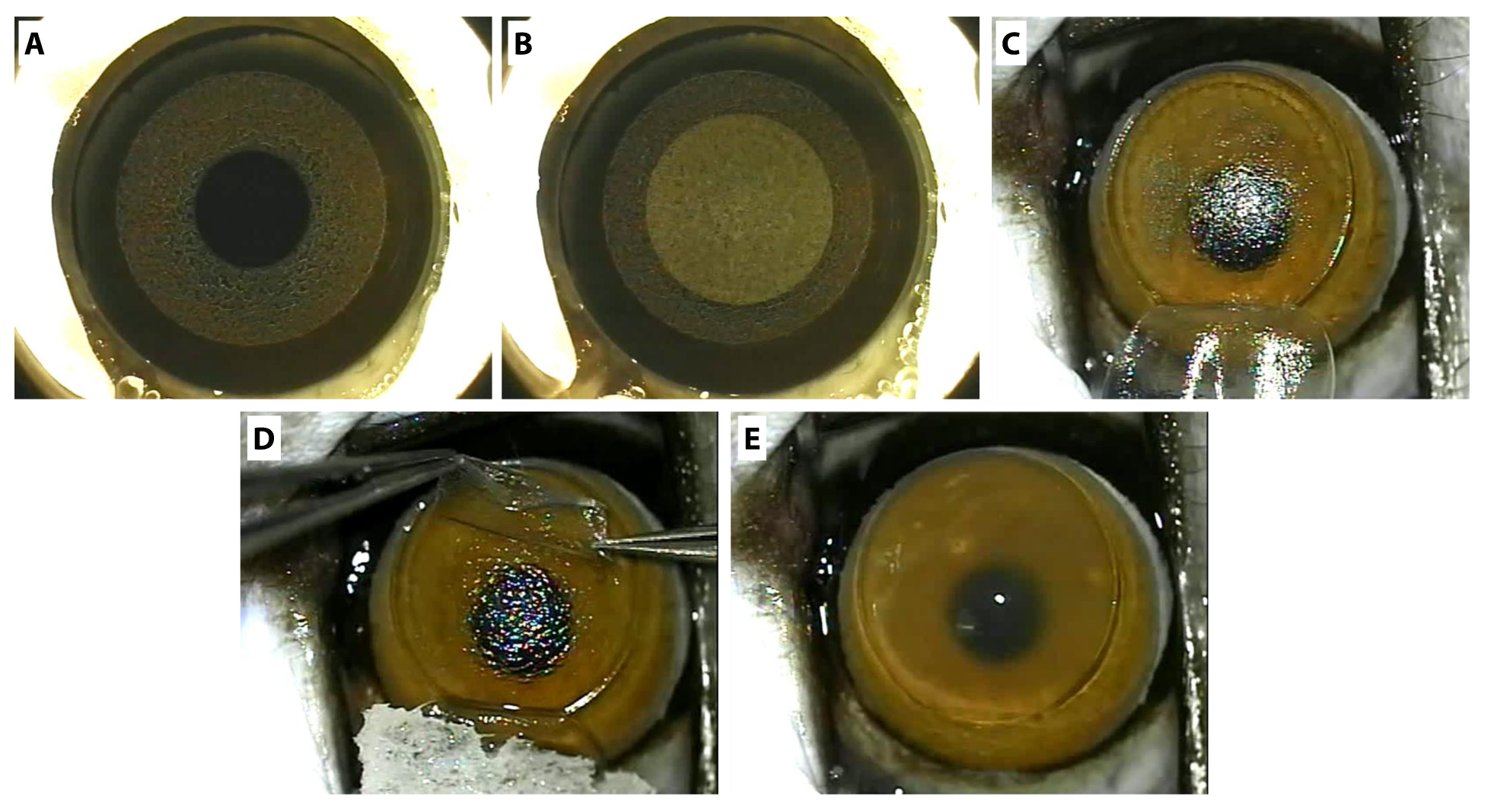

Supplement: Figure S1 — Photomontage of refractive lenticule extraction (ReLEx) procedure. After fixation of cornea under the suction cone, the femtosecond laser first creates the posterior surface of the lenticule centripetally (A), followed by the anterior surface of the lenticule centrifugally (B). The resultant anterior flap is then lifted (C), similar to a LASIK flap, and the refractive lenticule is manually removed (D). The flap is finally repositioned (E). (TIF) [file pone.0067058.s001.tif]

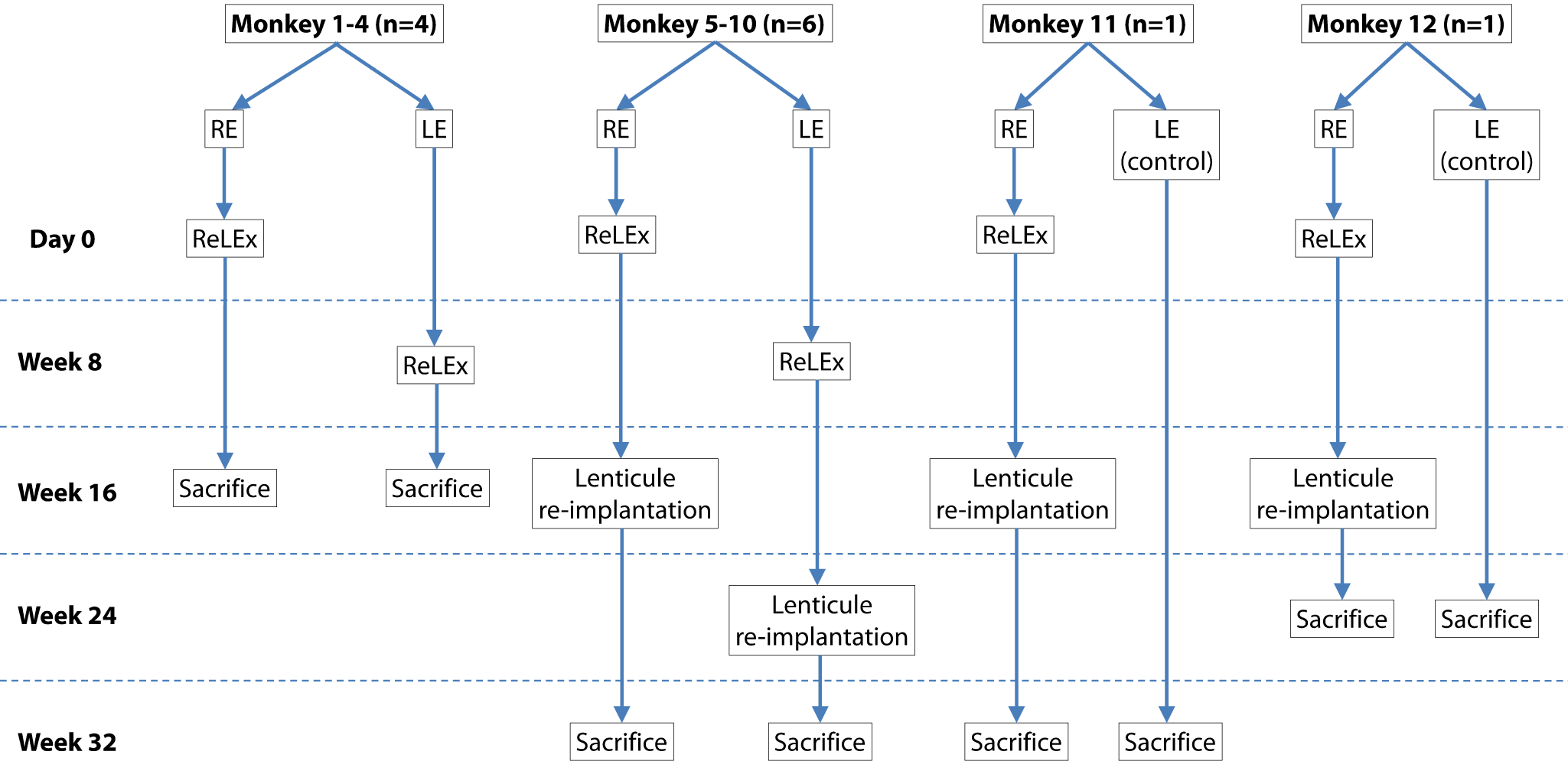

Supplement: Figure S2 — Flowchart showing the time points at which the samples were collected: ReLEx or lenticule re-implantation was performed during the study. RE and LE denote right eye and left eye, respectively. ReLEx is the abbreviation of Refractive Lenticule Extraction. (TIF) [file pone.0067058.s002.tif]

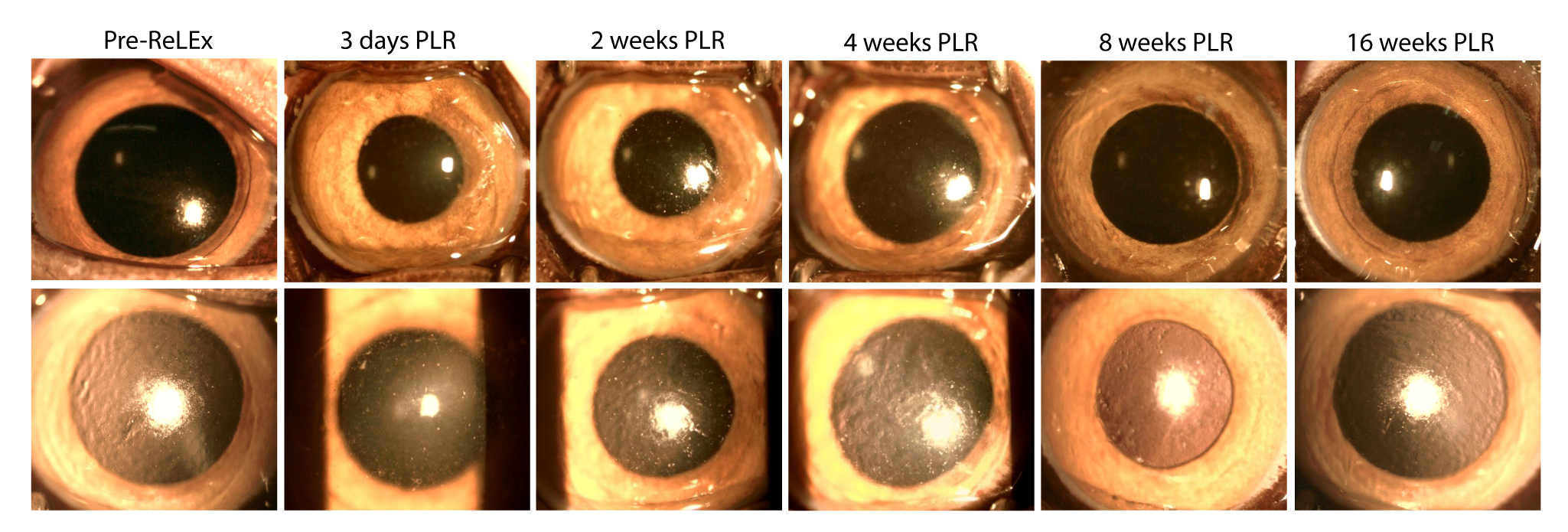

Supplement: Figure S3 — Slit lamp and retro illumination photographs of the post-reimplantation corneas. Slit lamp (top panel) and retro illumination photographs (bottom panel) of the cornea before ReLEx and on day 3, week 2, 4, 8 and 16 after refractive lenticule re-implantation. On day 3, the cornea appeared hazy with appearance of post-surgical debris. The appearance of haze was reduced in the subsequent follow-ups and was absent by week 8 and 16. PLR: post-lenticule re-implantation. (TIF) [file pone.0067058.s003.tif]

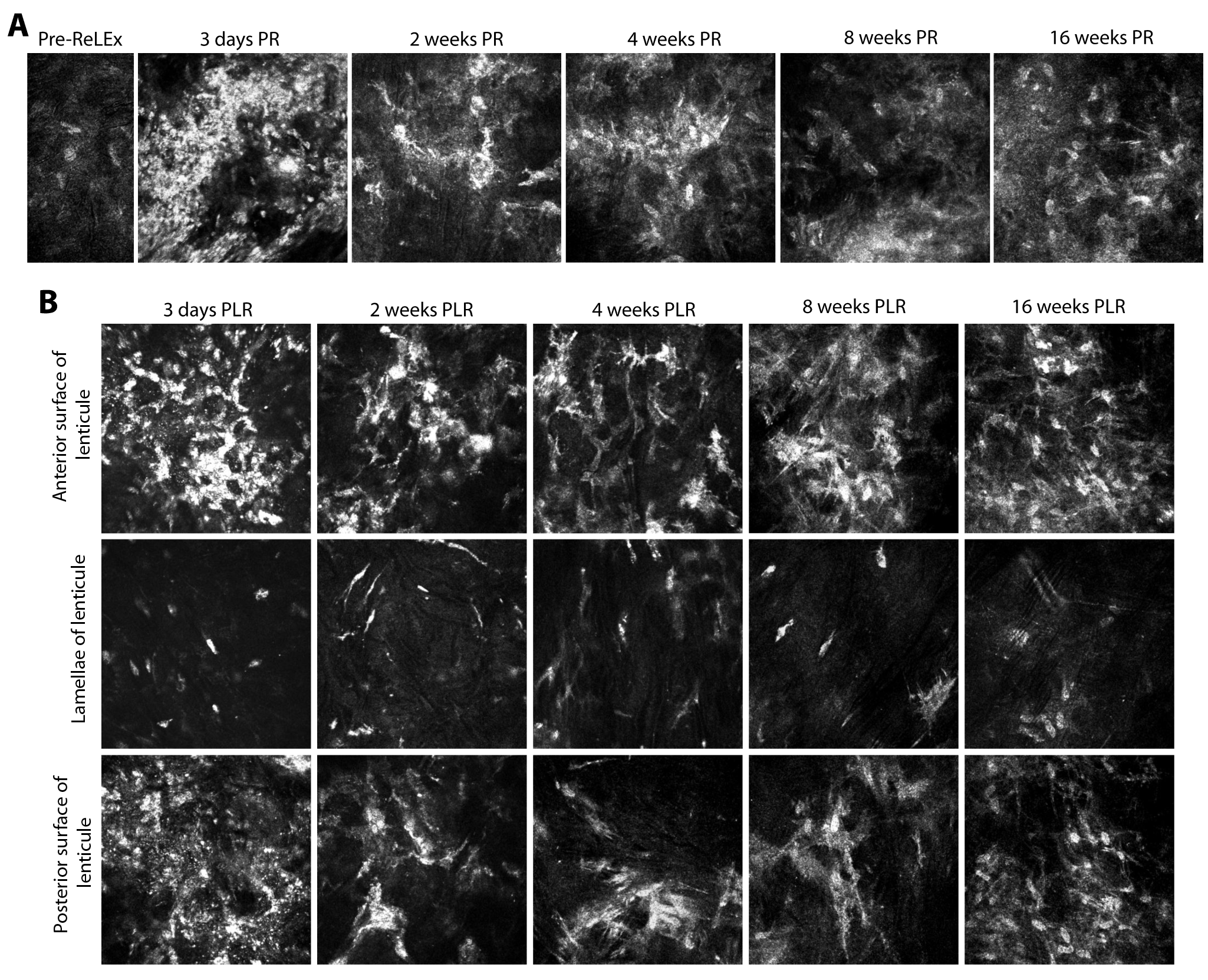

Supplement: Figure S4 — In vivo confocal micrographs of the pre- and post-operative corneas. (A) Horizontal surgical plane between the flap and stromal bed on day 3, weeks 2, 4, 8 and 16 after ReLEx. This region is normally marked by the presence of light reflective particles (haze), which gradually decreased in intensity over time. Keratocyte re-population could be observed on week 8 and 16. (B) The top panel shows the anterior interface of the re-implanted refractive lenticule. The middle panel shows the presence of keratocytes within the lamellae of the lenticule and the bottom panel shows the posterior interface of the lenticule. The intensity of the reflective layer observed in both interfaces was attenuated over time. Keratocyte re-population of anterior and posterior borders of the lenticule occurred by week 8 after lenticule re-implantation. PR: post-ReLEx, PLR: post-lenticule re-implantation. (TIF) [file pone.0067058.s004.tif]
